# Insights into vaccination: a cross-sectional study of knowledge, attitudes, and barriers among community pharmacists in Türkiye

**DOI:** 10.3389/fpubh.2025.1697400

**Published:** 2026-01-14

**Authors:** Nilay Aksoy, Zekiye Yılmaz, Nur Ozturk, Merve Kocyigit, Isıl Ozoglu

**Affiliations:** 1 Department of Clinical Pharmacy, Faculty of Pharmacy, Altınbaş University, İstanbul, Türkiye; 2 Department of Clinical Pharmacy, Faculty of Pharmacy, Acibadem Mehmet Ali Aydinlar University, Istanbul, Türkiye; 3 Clinical Pharmacy PhD Program, Graduate School of Health Sciences, Istanbul Medipol University, İstanbul, Türkiye

**Keywords:** vaccination, community pharmacists, knowledge, attitudes, public health

## Abstract

**Background:**

Community pharmacists play a vital role in public health by promoting and providing vaccination services. Their knowledge, attitudes, and perceived barriers are critical determinants of their effectiveness in this role. The primary outcomes of this study were pharmacists’ knowledge, attitudes, and logistical challenges related to vaccination, with the hypothesis that these factors differ according to sex, years of experience, job title, and pharmacy location.

**Materials and methods:**

This study is an online cross-sectional survey of all community pharmacists. A standardized 50-item questionnaire was used to obtain demographic information, vaccination knowledge, attitudes toward vaccines, and barriers to vaccination. Descriptive statistics such as frequencies, percentages, means, medians, standard deviations and chi-square tests were applied via SPSS 29.0 to analyze the dataset.

**Results:**

An online survey of 489 pharmacists revealed critical findings. Significant knowledge gaps exist, particularly concerning tetanus vaccination: only 59.3% knew the booster dose, and 54.4% recognized the primary 3-dose series. Attitudinally, 18% were ambivalent or did not advocate the influenza vaccine. Demographic analyses revealed complex influences: female pharmacists were less likely to agree that vaccines are safe (OR = 0.57, *p* = 0.0107) and less likely to feel professional pressure (OR = 0.54, *p* = 0.0043) than males were. Critically, the perception that tetanus is a serious threat was negatively correlated with age (*r* = −0.2390, *p* < 0.001). Major systemic barriers include the lack of authority to administer vaccines, insufficient reimbursements (68.5% reporting inadequacy), and the widespread absence of a method to identify unvaccinated adults (91% reporting no method).

**Conclusion:**

Before advocating for an expanded scope to include vaccine administration, substantial efforts to improve pharmacists’ knowledge and attitudes and address logistical barriers must be prioritized.

## 1 Introduction

Vaccination is one of the most successful and cost-effective public health interventions, saving millions of lives annually. It improves global health by reducing the incidence of infectious diseases and their associated sequelae (1). Vaccination protects vaccinated individuals and provides population-wide protection through herd immunity and decreased disease incidence. However, the success of vaccination programs is strongly dependent on adequate vaccine uptake and high coverage rates in communities (2). Building on these principles, Türkiye’s experience shows that the impact of vaccine programs varies across populations. Childhood coverage is generally high and similar to OECD/EU averages, whereas adult uptake, especially for influenza and pneumococcal vaccines and COVID-19 booster coverage, tends to be lower than that in much of Western Europe (3–5). In countries where pharmacies play a greater role in adult vaccination, such as the United Kingdom, Portugal, and Ireland, uptake among priority groups is greater, suggesting that expanding pharmacist involvement could help improve adult coverage in Türkiye (6, 7).

Community pharmacists can help increase vaccination rates by offering accessible and convenient vaccine delivery methods. They have effectively carried out these responsibilities in community pharmacy settings globally, providing year-round vaccinations (3). Community pharmacy-based vaccination services (CPBVS) are essential in bridging knowledge and vaccine administration gaps. In addition, these services are less expensive for individuals and insurance companies, and they can increase vaccination rates (8). However, the execution of CPBVS has faced many obstacles, such as issues involving the accessibility of secure premises for vaccine administration, payment procedures, doctor assistance, community pharmacist (CP) expertise and ability levels, public demand and trust, educational programs, policies, patient safety concerns, understanding within the health care sector, individuals’ resistance, shortages of staff, and adapting to accommodate the unique situation (9).

Moreover, considering the COVID-19 pandemic, it has become evident that pharmacists must have explicit knowledge of vaccination procedures (10). However, the extent to which CPs may contribute to vaccination remains to be determined since their knowledge, attitudes, and practices about vaccination differ substantially. For example, many knowledge gaps have been highlighted, including unawareness of specific vaccine recommendations, contraindications, and adverse events (11, 12). However, even more significance should be attached to personal beliefs, perceived barriers, and logistical issues in determining the readiness and capacity for pharmacy vaccination (13, 14).

Given the significance of further education for community pharmacists, removing potential obstacles and maximizing their vaccination potential, it is critical to comprehend their attitudes and knowledge in this area fully. The urgency and necessity of continuous learning in the field of pharmacy cannot be overstated. Although multiple studies have examined pharmacists’ vaccination-related knowledge, attitudes, and practices across diverse settings, their conclusions are mixed. In Italy, Della Polla et al. reported that community pharmacists generally endorsed vaccination but reported knowledge gaps for specific vaccines and target groups, limited prior training, and variability in the recommendation or administration of vaccines, with legal and organizational constraints as key barriers (11). Surveys in other contexts likewise show supportive attitudes but variable knowledge and uneven implementation, shaped by training, workflow, regulatory scope, reimbursement, and interprofessional perceptions (12, 13). This variability is further illuminated by qualitative findings, which point to persistent barriers, including time constraints, staffing, confidence in addressing hesitancy, and health-system linkages and facilitators such as accessibility and clear referral pathways (14).

These patterns, observed across both quantitative and qualitative studies, point to a broader methodological challenge: heterogeneous contexts, nonstandardized outcomes, and dependence on cross-sectional or preliminary evidence limit comparability and generalizability. Consequently, there remains a clear need for methodologically robust, setting-specific data tailored to local regulatory environments and population needs.

Pharmacists are legally licensed to administer particular vaccines in the United States, Canada, and Australia. In several European countries, such as France, Norway, Portugal, and Switzerland, pharmacists are authorized to administer a comprehensive range of vaccines, including those in national immunization schedules and travel vaccines. In contrast, other European countries, including Belgium, Denmark, Finland, Germany, Greece, Ireland, Italy, Latvia, Lithuania, Luxembourg, Poland, and Romania, currently allow pharmacists to administer a narrower set of vaccines, most commonly influenza and COVID-19. Pharmacist-led vaccination is under policy consideration but not yet implemented in Croatia, Estonia, Hungary, India, Malta, Serbia, Slovenia, Tanzania, Türkiye, and Uruguay, where authorities are actively evaluating regulatory changes and engaging in ongoing policy deliberations and advocacy. Pharmacists are not authorized to administer vaccines in Sweden, Ukraine, the Netherlands or several other countries, where no explicit legal framework currently permits pharmacist-administered vaccination. Iceland is piloting pharmacist-administered vaccination in two pharmacies to assess feasibility and impact, and Singapore launched a Ministry of Health trial on 28 October 2024 allowing flu vaccinations by pharmacists in three pharmacies (3, 15).

Notably, international studies and reviews confirm that pharmacy-based immunization services reduce healthcare costs by lowering primary care visits, hospitalizations, and productivity losses (16, 17).

However, CPs in Türkiye are not permitted to administer vaccines in their pharmacies (15). Prior research on CP vaccination knowledge, attitudes, and practices for pharmacists in Türkiye revealed that the majority wanted the authority to vaccinate. They expected that this new responsibility would improve patient access to vaccinations, resulting in higher vaccination rates (12). Although CPs may not have the authority to administer vaccinations, they can still provide valuable services to increase vaccination rates. In addition to their logistical involvement in supplying the vaccine, their role in educating and raising awareness about vaccines, their effects, side effects, and related topics is crucial. However, CPs must possess sufficient knowledge and a positive attitude toward vaccination to do so effectively.

This study aimed to assess community pharmacists’ vaccination-related knowledge, attitudes, and logistical barriers, focusing on awareness and agreement with current recommendations and perceptions of supply chain factors. The primary outcomes were knowledge, attitudes, and logistics-related challenges; the key independent variables were gender, years of experience, job title, and pharmacy location. We hypothesized that these outcomes would differ across these demographic and practice characteristics. The findings are intended to inform targeted interventions to strengthen the role of pharmacists in vaccination services and improve public health.

## 2 Materials and methods

### 2.1 Study design

A descriptive, cross-sectional observational study examined community pharmacists’ knowledge of and attitudes toward vaccination. The researchers issued an anonymous online survey to the respondents via Google Forms.

### 2.2 Study instrument

The survey tool, consisting of 50 questions organized into several sections, was developed by the research team via items from the literature (13) and translated from English to Turkish. The translation and back-translation process followed the World Health Organization (WHO) guidelines for the translation and adaptation of instruments (18) and was subsequently reviewed by two clinical pharmacists (NA and ZY) and refined on the basis of pre-survey results. The questions sought to understand the demographic attributes, knowledge, and approach of vaccination recommendations, plus the supply chain and logistics management for the vaccination services. The questionnaires covered the demographic profile of the participants and assessed their age, sex, job title, years of experience in community pharmacies, and pharmacy location. Additionally, the survey considered the knowledge and approach response to vaccination provided a scenario of a pharmacist’s rated knowledge of proper vaccination as recommended by the Türkiye immunization advisory board, and agreed with its recommendation of immunization for various samples of the vaccine, such as influenza, pneumonia, and tetanus. The supply chain and logistics section included how unvaccinated patients are identified; pressure from one’s professional organization; information availability; adverse events reported; barriers experienced, including complexity, time burdens, and record-keeping; and the adequacy of compensation.

### 2.3 Participant recruitment

The sample size was determined on the basis of the primary objective of estimating key proportions related to pharmacists’ vaccination knowledge, attitudes, and logistical barriers with adequate precision. Assuming a conservative expected proportion of 50% (which maximizes the required sample size in the absence of prior estimates), a 95% confidence level (*Z* = 1.96), and a 5% absolute margin of error (d = 0.05), the initial sample size was calculated via Cochran’s formula for a single proportion: $n0=\frac{Z^{2}\cdot p\cdot (1-p)}{d^{2}}$ . Applying the finite population correction for the target population of community pharmacists in Türkiye (*N* = 28,465) yielded n = n0 / [1 + (n0–1)/N] ≈ 381. The survey link, accompanied by a brief study introduction and assurances of anonymity and confidentiality, was emailed to 6,100 of the 28,465 community pharmacists in Türkiye. The survey was administered online via Google Forms. Pharmacists who agreed to participate provided informed consent and then completed the questionnaire. In total, 500 pharmacists submitted a survey (overall response rate: 500/6,100 = 8.2%). Of these, 11 submissions were excluded because of missing data, resulting in 489 (97.8%) complete questionnaires.

### 2.4 Data collection, procedure, and analysis

#### 2.4.1 Instrument development and pilot testing

The questionnaire was developed by the research team on the basis of the literature and expert review. Prior to dissemination, the instrument was pilot-tested with 30 community pharmacists to assess its clarity, relevance, and internal consistency. These 30 pharmacists were not included in the main study population or analyses. Reliability was evaluated via Cronbach’s alpha for the multi-item scales; the alpha coefficient (0.727) indicated acceptable internal consistency.

#### 2.4.2 Data capture and procedure

The finalized online questionnaire was administered via Google Forms and distributed to community pharmacists via email during the study period. Demographic variables (age, sex, job title, years of experience, pharmacy location) were recorded as self-reported. The knowledge variables reflected awareness of Immunization Advisory Board recommendations (influenza, pneumococcal, tetanus) and, where applicable, agreement with these recommendations. Attitudinal variables were derived from Likert-scale items on vaccine safety and effectiveness, perceived disease risks (influenza, pneumonia, tetanus), and trust in scientific knowledge. Supply chain/logistics variables included the presence of mechanisms to identify unvaccinated adults, perceived professional pressure, the adequacy of information/resources, adverse event reporting, perceived complexity and time burden, record-keeping barriers, reimbursement sufficiency, and the influence of practice logistics on providing vaccines.

#### 2.4.3 Data management

Data were captured in a secure online database upon submission and exported after database lock for analysis.

#### 2.4.4 Statistical analysis

Analyses were conducted via SPSS version 29.0 (IBM, 2022). Descriptive statistics (frequencies, percentages, means, medians, interquartile ranges, and standard deviations) were calculated for demographics and survey items. Bivariate analyses: Group differences across key demographics (gender, job title, years of experience, and pharmacy location) for categorical outcomes (knowledge, attitudes, beliefs, and perceptions of vaccination services) were assessed via chi-squared tests. The comprehensive analysis used three primary statistical techniques, chi-square tests, Spearman’s rank correlation, and binary logistic regression to understand the relationships between the demographic variables of the pharmacists and their attitudes toward vaccination. Statistical significance was defined as a two-sided *p* value < 0.05.

### 2.5 Ethical consideration

Ethical approval for the study protocol was provided by the Research Ethical Committee of Altınbaş University (Approval No. 2023/224). The survey was conducted voluntarily, and informed consent was provided for all the participants who were required to confirm it before proceeding with the questionnaire. No personal identifiers were gathered, preserving the anonymity of the collected responses. The data were stored securely by the research team and were accessible to the team members only.

## 3 Results

Four hundred eighty nine participants, with a mean age of 37.78 years (SD = 10.34), were included in the study. Most participants were female; 59.3 and 85.5% of the population were responsible for pharmacists. A total of 30.3% had less than 5 years of experience, and most worked in pharmacies located in local neighborhoods. The basic demographics and general practice statistics are summarized in Table 1. The vast majority of the pharmacists agreed that vaccines are effective (87.1%) and safe (87.1%). 98% of pharmacists dispense vaccines to adults. The most common indication for adult vaccination is routine (59.9%). The two most significant barriers were insufficient reimbursements (68.5% ‘Disagree’/‘Neither agree nor disagree’ that they are sufficient) and obstacles related to vaccine record-keeping (35.8% ‘Agree’ which is a barrier). The largest percentage of respondents (54%) disagreed that vaccines are too time-consuming to dispense. A large majority (91%) do not have a method for identifying unvaccinated adult patients. Table 2 presents key attitudes and knowledge regarding specific vaccines.

**Table 1:** Demographic characteristics and general vaccine practice.

Variables	Median	Mean	IQR	SD
Age	36	37.78	29–45	10.364
			**N**	**%**
Gender	Male		199	40.7
	Female		290	59.3
Job Title	Pharmacist		418	85.5
	Second Pharmacist		19	3.9
	Assistant Pharmacist		52	10.6
How long have you been in community pharmacy?	<5 years		148	30.3
	5- < 10 years		93	19
	10- < 20 years		114	23.3
	20 + years		134	27.4
Where is your pharmacy located?	Around the hospital		135	27.6
	On the busy street of the district		117	23.9
	In the shopping mall		5	1
	In a local neighborhood		232	47.4
Does your pharmacy provide vaccinations for children, adolescents and young people?	Yes		439	89.8
	Yes, only to those 5 and older		15	3.1
	No		35	7.2
If yes, for what indications?	Routine		393	80.4
	High-risk		53	10.8
	Travel		3	0.6
	Research		5	1
Approximately how many vaccinations on average do you dispense per month for children, adolescents, and youth?	<1 vaccine		135	27.6
	1–5 vaccines		227	46.4
	6–10 vaccines		60	12.3
	11–20 vaccines		24	4.9
	21–50 vaccines		3	0.6
	51 + vaccines		5	1
Do you dispense vaccines to adults?	Yes		479	98
	No		10	2
If yes, for what indications?	Routine		293	59.9
	High-risk		167	34.2
	Travel		12	2.5
	Research		7	1.4
Approximately how many vaccinations on average do you dispense per month to adults?	<1 vaccine		136	27.8
	1–5 vaccines		226	46.2
	6–10 vaccines		66	13.5
	11–20 vaccines		25	5.1
	21–50 vaccines		16	3.3
	51 + vaccines		10	2
Do you have a method for identifying unvaccinated adult patients?	Yes		44	9
	No		445	91

**Table 2:** Pharmacist knowledge, attitudes, and barriers.

Survey question	Response	*N*	%
Vaccines are generally safe.	Disagree	16	3.3
	Neither agree nor disagree	47	9.6
	Agree	426	87.1
Flu poses serious health risks to adults.	Disagree	16	3.3
	Neither agree nor disagree	111	22.7
	Agree	362	74.0
Pneumonia poses a serious threat to individuals over the age of 65.	Disagree	22	4.5
	Neither agree nor disagree	72	14.7
	Agree	395	80.8
I trust the current scientific knowledge about vaccines.	Disagree	281	57.7
	Neither agree nor disagree	135	27.6
	Agree	73	14.9
	Neither agree nor disagree	143	29.2
	Disagree	188	38.4
Vaccines are too time consuming to dispense.	Agree	93	19
	Neither agree nor disagree	132	27
	Disagree	264	54
Obstacles related to vaccine record-keeping are a significant barrier to vaccine dispense.	Agree	175	35.8
	Neither agree nor disagree	162	33.1
	Disagree	152	31.1
Reimbursements for vaccine dispense are sufficient.	Agree	66	13.5
	Neither agree nor disagree	88	18
	Disagree	335	68.5

Table 3 summarizes how various demographic factors influence pharmacists’ vaccine-related practices and attitudes, presenting the results of a chi-square analysis. Statistically significant associations were found between demographic factors and several pharmacist attitudes and practices related to vaccination. Compared with male pharmacists, female pharmacists were significantly less likely to agree that “Vaccines are generally safe” (69.66% vs. 80.40%, *p* = 0.015). However, females were more aware of the pneumonia vaccine recommendation for healthy adults over 65 years of age (84.48% vs. 75.88% of males, *p* = 0.017). With respect to logistical barriers, younger pharmacists (those with <5 years of experience) were more likely to perceive obstacles related to vaccine record-keeping as a significant barrier (38.51% agreed) than were those with >20 + years of experience (35.82% agreed, *p* = 0.047). Location also played a role, as a significantly greater percentage of pharmacists working in the shopping mall (40.00%) agreed that flu is so rare that adults no longer need to be vaccinated, than those around the hospital (4.44%, *p* = 0.013). Finally, Responsible Pharmacists demonstrated a greater degree of agreement (91.87%) that “Individuals over the age of 65 are at risk of developing pneumonia,” compared with Second Pharmacists (78.95%, *p* = 0.003).

**Table 3:** Influence of demographics on practice and attitudes.

Influences by the gender of the participants				
Question	Answer	Gender		*p* value
		Male	Female	
Vaccines are generally safe.	Disagree	7 (3.51%)	9 (3.10%)	0.015
	Neither agree nor disagree	32 (16.08%)	79 (27.24%)	
	Agree	160 (80.40%)	202 (69.66%)	
Flu poses serious health risks to adults.	Disagree	23 (11.56%)	33 (11.38%)	0.032
	Neither agree nor disagree	36 (18.09%)	82 (28.28%)	
	Agree	140 (70.35)	175 (60.34%)	
Are you aware that the Immunization Advisory Board recommends that healthy 65 years of age or older adults receive a vaccine for protection against Pneumonia?	Yes	151 (75.88%)	245 (84.48%)	0.017
	No	48 (24.12%)	45 (15.52%)	
Do you have a method for identifying unvaccinated adult patients?	Yes	25 (12.56%)	19 (6.55%)	0.022
	No	174 (78.44%)	271 (93.45%)	
I feel under professional pressure to dispense vaccines.	Agree	62 (31.16%)	57 (19.66%)	0.005
	Neither agree nor disagree	55 (27.64%)	75 (25.86%)	
	Disagree	82 (41.20%)	158 (54.48%)	
Vaccines are too time-consuming to dispense.	Agree	48 (24.12%)	45 (15.52%)	0.021
	Neither agree nor disagree	57 (27.64%)	75 (25.86%)	
	Disagree	94 (47.24%)	170 (58.62%)	
Obstacles related to vaccine record-keeping are a significant barrier to vaccine dispense.	Agree	67 (33.67%)	108 (37.24%)	0.017
	Neither agree nor disagree	80 (40.20%)	82 (28.28%)	
	Disagree	52 (26.13%)	100 (34.48%)	
Reimbursements for vaccine dispense are sufficient.	Agree	33 (16.58%)	33 (11.38%)	0.017
	Neither agree nor disagree	25 (12.56%)	63 (21.72%)	
	Disagree	141 (70.86%)	194 (66.90%)	
Practice logistics are a factor in deciding whether or not to give vaccines.	Agree	89 (44.72%)	122 (42.07%)	0.008
	Neither agree nor disagree	61 (30.65%)	123 (42.41%)	
	Disagree	49 (24.62%)	45 (15.52%)	
I support expanding the scope of practice of pharmacists to include providing vaccinations to adults.	Agree	176 (88.94%)	231 (79.31%)	0.003
	Neither agree nor disagree	18 (9.05%)	28 (9.66%)	
	Disagree	5 (2.51%)	31 (10.69%)	
I support expanding the scope of practice of pharmacists to include providing vaccinations to children and adolescents (ages 5 to 18).	Agree	161 (80.91%)	217 (74.83%)	0.012
	Neither agree nor disagree	25 (12.56%)	29 (10.00%)	
	Disagree	13 (6.53%)	44 (15.17%)	
Pharmacists should be allowed to vaccinate children under 5 years of age.	Agree	64 (32.16%)	54 (18.62%)	0.001
	Neither agree nor disagree	40 (20.10%)	55 (18.97%)	
	Disagree	95 (47.74%)	181 (62.41%)	

Influences by the year of experience						
Question	Answer	<5 year	5- < 10 year	10- < 20 year	20 + year	
Tetanus poses a serious threat to adult health.	Disagree	13 (8.78%)	3 (3.23%)	20 (17.54%)	33 (24.63%)	<0.001
	Neither agree nor disagree	32 (21.62%)	19 (20.43%)	27 (23.68%)	37 (27.61%)	
	Agree	103 (69.59%)	71 (76.34%)	67 (58.77%)	64 (47.76%)	
The adult population is at significant risk of getting tetanus.	Disagree	19 (12.84%)	15 (16.13%)	33 (28.95%)	40 (29.85%)	<0.001
	Neither agree nor disagree	59 (39.86%)	29 (31.18%)	44 (38.60%)	51 (38.06%)	
	Agree	70 (47.30%)	49 (52.69%)	37 (32.46%)	43 (32.09%)	
Tetanus is so rare that the adult population no longer needs to be vaccinated against it.	Disagree	101 (68.24%)	53 (54.14%)	58 (50.88%)	69 (51.49%)	0.035
	Neither agree nor disagree	33 (22.30%)	25 (26.88%)	39 (34.21%)	38 (28.36%)	
	Agree	14 (9.46%)	15 (16.13%)	17 (14.91%)	27 (20.15)	
Obstacles related to vaccine record-keeping are a significant barrier to vaccine dispensing.	Disagree	36 (24.32%)	27 (26.21%)	36 (31.58%)	53 (39.56%)	0.047
	Neither agree nor disagree	55 (37.16%)	39 (37.86%)	45 (39.47%)	33 (24.62%)	
	Agree	57 (38.51%)	37 (35.92%)	33 (28.95%)	48 (35.82%)	

Influences by the pharmacy location						
Question	Answer	Near hospital	On a busy street	At the shopping mall	In a local neighborhood	*p* value
Flu is so rare that the adult population no longer needs to be vaccinated.	Disagree	110 (81.48%)	98 (83.76%)	2 (40.00%)	185 (79.31%)	0.013
	Neither agree nor disagree	19 (14.07%)	15 (12.82%)	1 (20.00%)	37 (15.95%)	
	Agree	6 (4.44%)	4 (3.42%)	2 (40.00%)	10 (4.31%)	
If yes, do you agree with this recommendation?	Disagree	76 (77.55%)	76 (86.36%)	5(100.00%)	132 (70.59%)	0.025
	Neither agree nor disagree	14 (14.29%)	5 (5.6%)	0 (0.00%)	42 (22.46%)	
	Agree	8 (8.16%)	7 (7.95%)	0 (0.00%)	13 (6.95%)	
Do you have a method for identifying unvaccinated adult patients?	Yes	13 (9.63%)	3 (2.56%)	1 (20.00%)	27 (11.64%)	0.034
	No	122 (90.37%)	114 (97.44%)	4 (80.00%)	205 (88.36%)	
Pharmacists have adequate training to supply the vaccine.	Disagree	10 (7.41%)	6 (5.13%)	1 (0.82%)	26 (11.21%)	0.017
	Neither agree nor disagree	13 (9.63%)	26 (22.22%)	0 (0.00%)	25 (10.78%)	
	Agree	112 (82.96%)	85 (72.65%)	54 (98.18%)	181 (78.00%)	

Influences by the position of the pharmacist					
Question	Answer	Responsible pharmacist	Second pharmacist	Assistant pharmacist	*p* value
Individuals over the age of 65 are at risk of developing pneumonia.	Disagree	10 (2.39%)	0 (0.00%)	5 (9.62%)	0.003
	Neither agree nor disagree	24 (5.74%)	4 (21.05%)	2 (3.85%)	
	Agree	384 (91.87%)	15 (78.95%)	45 (86.54%)	
Pneumonia is so rare that people over the age of 65 no longer need to be vaccinated.	Disagree	377 (90.22%)	15 (78.95%)	41 (78.85%)	0.002
	Neither agree nor disagree	31 (7.42%)	4 (21.05%)	5 (9.62%)	
	Agree	10 (2.39%)	0 (0.00%)	6 (11.54%)	
If yes, do you agree with this recommendation?	Disagree	304 (90.48%)	10 (66.67%)	39 (92.86%)	0.022
	Neither agree nor disagree	19 (5.65%)	4 (26.67%)	1 (2.38%)	
	Agree	13 (3.87%)	1 (6.67%)	2 (4.76%)	
My colleagues think it is important for us to provide vaccines.	Disagree	11 (2.6%)	1 (5.26%)	2 (3.8%)	0.004
	Neither agree nor disagree	51 (12.2%)	8 (42.11%)	6 (11.5%)	
	Agree	(2.6%)	10 (52.63%)	44 (84.6%)	
The increasing number of vaccines makes it too complicated to provide them.	Disagree	170 (40.74%)	4 (21.05%)	13 (26.9%)	0.041
	Neither agree nor disagree	122 (29.12%)	4 (21.05%)	17 (32.7%)	
	Agree	126 (30.14%)	11 (57.89%)	21 (40.4%)	
I support expanding the scope of practice of pharmacists to include providing vaccinations to children and adolescents (ages 5 to 18).	Disagree	52 (12.4%)	0 (0.00%)	5 (9.6%)	0.024
	Neither agree nor disagree	39 (9.3%)	5 (26.3%)	10 (19.2%)	
	Agree	327 (78.2%)	14 (73.7%)	37 (71.2%)	

Spearman’s correlation analysis (Table 4) revealed that a pharmacist’s age significantly correlated with several vaccine-related attitudes. A negative correlation was found between age and the belief that tetanus poses a serious threat to adult health (*r* = −0.2390, *p* < 0.001) and that the adult population is at significant risk of developing tetanus (*r* = −0.1929, *p* < 0.001). Conversely, a positive correlation was observed between age and the belief that tetanus is so rare that the adult population no longer needs to be vaccinated against it (*r* = 0.1408, *p* = 0.0018). Additionally, the perception of obstacles related to vaccine record-keeping as a significant barrier was negatively correlated with age (*r* = −0.102, *p* = 0.024).

**Table 4:** Correlations between pharmacist age and vaccine-related attitudes.

Attitude question	Correlation (r)	*p*-value
Tetanus poses serious threat to adult health.	−0.2390	<0.001
The adult population is at significant risk of getting tetanus.	−0.1929	<0.001
Tetanus is so rare that the adult population no longer needs to be vaccinated against it.	0.1408	0.0018
Flu poses serious health risks to adults.	0.1096	0.0153
Obstacles related to vaccine record-keeping are a significant barrier to vaccine dispense.	−0.102	0.024

Logistic regression analysis (Table 5) was used to explore the predictors of core attitudes further. Being a female pharmacist (versus male) was found to be a significant negative predictor of two attitudes: the belief that vaccines are safe (odds ratio (OR) = 0.57, *p* = 0.0107) and the feeling of being under professional pressure to dispense vaccines (OR = 0.54, *p* = 0.0043). Furthermore, being a responsible pharmacist (versus other job title) was a positive predictor of the belief that the flu poses serious health risks to adults (OR = 1.83, *p* = 0.0292).

**Table 5:** Logistic regression odds ratios (ORs) for core attitudes*.

Dependent variable (Attitude)	Significant predictor (X)	Odds ratio (OR)	*p*-value
Vaccine are safe	Gender: Female versus male	0.57	0.0107
I feel under professional pressure to dispense vaccines.	Gender: Female versus male	0.54	0.0043
Flu poses serious health risks to adults.	Job: Responsible Pharmacists various others.	1.83	0.0292

*This analysis focused on three core attitude questions, vaccine is effective, vaccine are safe, vaccine is time consuming. The Chi-Square analysis found no statistically significant associations (*p* < 0.05) between these three core attitude and other variables.

## 4 Discussion

This study provides valuable insights into the demographics, general vaccine practices, attitudes, and correlates of vaccine-related perceptions among community pharmacists in Türkiye. The findings collectively suggest a high level of engagement in and support for expanded vaccination services, although they also highlight specific demographic influences and attitudinal concerns that warrant consideration.

The survey sample, with a majority of female participants, aligns with the increasing representation of females in the pharmacy workforce globally (19, 20). Most surveyed community pharmacies reported dispensing vaccines to children, adolescents, and adults. However, a low volume was observed, with 46.2% dispensing only 1–5 adult vaccines per month. The low volume observed is likely a result of the collective impact of many systemic barriers, especially the insufficient reimbursement and possible restrictions on the pharmacist’s authority to fully administer (inject) the vaccine in the Turkish context. Turkish regulations limit pharmacists to only dispensing (selling) vaccines rather than administering the injection, patients must still seek out a separate healthcare provider for the shot (15). This restriction eliminates the convenience of a “one-stop-shop” service, which significantly decreases the likelihood of patients completing the process at the pharmacy and thus limits the vaccination volume. Furthermore, inadequate reimbursement, administrative costs, and the lack of effective delivery programs collectively discourage pharmacists from prioritizing vaccination activities in their workflow (21). International research shows that inconsistent and insufficient compensation for pharmacists is a common barrier globally, limiting their ability to proactively promote vaccination. In many settings, pharmacists are only reimbursed for the act of administering the vaccine, not for the time spent on patient education or outreach, further reducing their motivation to integrate vaccination into routine practice (7, 22).

### 4.1 Knowledge and attitudes toward vaccines

The surveyed pharmacists generally held positive attitudes toward vaccine safety and the seriousness of preventable diseases. An overwhelming majority agree that vaccines are generally safe and 74.0% agree that the flu poses serious health risks to adults. Multiple studies across different countries have reported that a majority of pharmacists express confidence in vaccine safety and efficacy. For example, over 90% of pharmacists in several surveys agreed that vaccines are rigorously tested and play a key role in preventing infectious diseases (23).

Conversely, the data revealed a critical and complex attitudinal challenge regarding trust in scientific knowledge. While the pharmacists generally agreed that vaccines are safe, a substantial portion expressed skepticism toward the current scientific knowledge about them: 57.7% disagreed with the statement “I trust the current scientific knowledge about vaccines,” and 27.6% neither agreed nor disagreed. This widespread professional skepticism, despite high personal conviction in vaccine safety, is a concerning finding that could undermine patient confidence and professional advocacy efforts. Recent systematic reviews and studies highlight that pharmacists often have unsatisfactory knowledge about vaccines, with notable deficiencies in understanding vaccine safety, indications, and disease complications. These knowledge gaps are accompanied by shortcomings in attitudes and beliefs about vaccine safety, and a lack of proactive vaccine promotion for patients (24).

Crucially, the study identified significant knowledge gaps, particularly concerning tetanus vaccination, only 59.3% knew of the tetanus booster dose, and only 54.4% recognized the primary tetanus vaccination as a 3-dose series. These knowledge deficits, corroborated by the literature showing pharmacists’ unawareness of specific vaccine recommendations and contraindications, indicate an urgent need for targeted educational programs. Erku suggested that CPs might benefit from more training to help them deal with erroneous information while educating people about vaccinations (25). Similarly, Alyahya et al. mentioned that CPs also had extensive knowledge gaps about the HPV vaccine, underscoring a general lack of an increased amounts or levels of formal and informal CP education materials and parent awareness (26). Studies from Ethiopia and Malaysia (8, 27) emphasized that knowledge gaps affect CPs’ willingness to administer vaccines and their confidence in their ability to handle vaccine hesitancy. However, Nigerian CPs possess an excellent knowledge of vaccination and can thus undertake this role with sufficient training (28). In Türkiye, Ozdemir reported that CPs had limited knowledge of vaccination during pregnancy, emphasizing the importance of education in improving their knowledge and attitudes (12). For example, other studies have shown that CPs present knowledge gaps regarding vaccines addressed to children and individuals with chronic conditions (29, 30). These knowledge deficits, corroborated by the literature showing pharmacists’ unawareness of specific vaccine recommendations and contraindications, indicate an urgent need for targeted educational programs.

With respect to the influenza vaccine, this study highlights a complex and somewhat concerning landscape regarding the attitudes of CPs’ toward the influenza vaccine. An enormous majority know the general recommendations for influenza vaccination, yet too few are ambivalent or even in open disagreement with guidelines for the influenza vaccination schedule. Provided that CPs are on the frontline of vaccination advocacy and consultation, this may pose some critical questions about the potential impact on public health.

Several studies have investigated community pharmacists’ attitudes toward the influenza vaccine. This inquiry could offer valuable insights into how these professionals view their role in promoting and administering this most critical vaccine. Pullagura et al. reported that Canadian pharmacists have a relatively positive attitude toward the flu vaccine (31). The CPs reported the willingness of pharmacists, enthusiasm, and positive attitudes to participate in vaccination services generally in other surveys (32, 33).

### 4.2 Influence of demographics on attitudes and practice

#### 4.2.1 Gender

The influence of gender on attitudes is complex. Female pharmacists were significantly less likely to agree that vaccines are generally safe (69.66% vs. 80.40% of males, *p* = 0.015) and less likely to feel under professional pressure to dispense vaccines (OR = 0.54, *p* = 0.0043). Research has consistently revealed that female healthcare workers, including pharmacists, are more likely to express vaccine hesitancy and concerns about safety than their male counterparts are (34–36). For example, in Qatar, female pharmacists and pharmacy technicians reported more negative emotional responses and lower agreement on vaccine safety (35). The lower perceived professional pressure among females may be linked to general findings about job perceptions. It is possible that, for female pharmacists, the pressure to *dispense* is outweighed by the stress of other daily tasks, or that they may manage their professional expectations differently than male colleagues do. Multiple studies from the U. S., Middle East, Europe, and other regions consistently reported that female pharmacists reported higher overall job and career satisfaction than male pharmacists did (37–39).

The survey revealed that males were more likely to agree that vaccines are too time-consuming to dispense, while also being more supportive of expanding the scope of practice for vaccinations for both adults and children aged >5 years of age. This highlights a potential dichotomy where male pharmacists are simultaneously more aware of the time constraints (logistical barriers) but more conceptually supportive of clinical expansion (professional aspiration). This tension is common, as pharmacists globally are willing to expand services but identify time constraints, workload, and insufficient compensation as primary barriers. Male pharmacists may view expansion as a path to greater clinical engagement and professional satisfaction, despite acknowledging the current logistical challenges (40, 42).

Notably, female pharmacists were more aware of the Immunization Advisory Board’s recommendation for healthy adults aged >65 years old to receive a pneumonia vaccine. This may reflect a greater focus on health maintenance guidelines or a stronger inclination toward patient counseling and preventive care, which is sometimes associated with traditional female professional roles (43, 44).

#### 4.2.2 Years of experience

As years of experience increased, the proportion of pharmacists who agreed that “Tetanus poses a serious threat to adult health” decreased significantly (*p* < 0.001). This negative correlation with age (*r* = −0.2390) and the decreasing perception of risk (*r* = 0.1408 for belief that it is too rare) suggests that senior pharmacists may require updates on current adult immunization schedules to counteract the perception that tetanus is no longer a pressing concern owing to low incidence rates. Research in pharmacy and healthcare settings has revealed that as years of experience increase, pharmacists are less likely to view tetanus as a significant threat to adult health. This may be due to their longer exposure to low incidence rates of tetanus, leading to the perception that the disease is rare and not a pressing concern for adults (45).

#### 4.2.3 Pharmacy location

Pharmacists serving residential and densely populated areas were more concerned about the flu as a health risk. This concern might suggest that pharmacists perceive the importance of the influenza vaccine on the basis of the work environment, probably due to the greater number of patients seeking advice and treatment for flu in these areas. However, despite the perceived severity of the flu, the stresses and challenges of vaccinating in a high-demand setting may erode the consistency of vaccine advocacy. This finding aligns with previous analyses in urban settings, which revealed superior patient satisfaction and vaccination rates than did less populated/rural-based pharmacists (46). Additionally, CPs in higher-density areas are more attuned to public health challenges. These pharmacists may have greater exposure to patients and health concerns, which could influence their perceptions.

#### 4.2.4 Job title

In Turkey, there are three job titles for CPs in pharmacies: responsible pharmacists, who are often the owners and most experienced; second pharmacists, who take over responsibility after the responsible pharmacists; and assistant pharmacists, who are fresh out of pharmacy school and must complete a year of training before opening their pharmacy. The job title also tended to play a role in pharmacists’ attitudes toward the influenza vaccine. The responsible pharmacists perceived the flu as a dangerous condition to the health of adults more than their counterparts did. Additionally, the second and assistant pharmacists reported that administering additional vaccines was a barrier to their duties, a comment that may indicate concern over the logistical burden of managing vaccine services as an additional responsibility. This perception may further lessen enthusiasm for actively advocating for the flu vaccine, especially given the role overload in demand. Similar findings were reported by Jarab et al., who reported that CPs with higher educational skills and responsibilities were more willing to provide vaccination services than others were (47).

## 4.3 Barriers to vaccine advocacy

This study identified significant logistical obstacles to the optimal delivery of vaccines by community pharmacies (CPs). The capacity to identify unvaccinated adults among their patient population needs substantial improvement, as only 9% of the pharmacists surveyed reported having a mechanism to determine which adult patients were not immunized. This finding is consistent with the work of Kulczycki et al., who determined that many pharmacists also struggle with maintaining durable patient vaccination status due to the poor integration of information systems (48). Additionally, 35.8% of the CPs in our study reported that the lack of a proper system for vaccination record maintenance was a significant hurdle. This challenge aligns with those presented in the study by Bluml et al., which reported that accessing state immunization information systems (IISs) is essential for pharmacists to efficiently recognize and meet unmet vaccination needs (49). Other significant barriers include the perceived complexity of vaccination services and inadequate reimbursement, with 68.5% of the pharmacists feeling that reimbursements were inappropriate. Similarly, other studies have shown that a lack of training, a lack of patient demand, a lack of private areas to provide vaccines, and a lack of adequate reimbursement are barriers to vaccination provision (9). Similar findings, along with lack of authorization, lack of physician support, lack of training on indications and contraindications, lack of time, and poor knowledge, were reported in studies conducted in Poland, the United States, and Canada (50, 52). These findings and the literature collectively underscore the urgent need to address staffing shortages, integrate health information systems, and enact legislation enabling greater CP autonomy and resource allocation.

## 4.4 Impact of public health campaigns

Specifically, the study revealed the necessity for educational campaigns and initiatives, open communication regarding fears and convictions, and systemic changes related to practicing logistics and record-keeping. Increasing pharmacists’ expertise and attitudes while eliminating existing practical hurdles would maximize their contribution to better vaccination perceptions and outcomes for public health. When there is a robust public health campaign, a significant proportion of pharmacists could support the promotion of the flu vaccine. Such campaigns underpinned the value of vaccination and equipped the pharmacists with what they needed regarding tools and information. Amburgh et al. reported successfully implementing a pharmacist-managed immunization campaign in a rural area, which increased influenza vaccination rates (53). Future studies should examine the impact of targeted educational enforcement and logistical support propositions on pharmacists’ knowledge, attitudes, and practices in various neighborhoods. A longitudinal design would also be beneficial for understanding the growth of the role of pharmacists in immunization and identifying emerging barriers and facilitators.

## 5 Limitations of the study

Several limitations to this study must be acknowledged. Self-reported data are subject to bias in that participants may overstate knowledge or underreport negative attitudes. The cross-sectional design provides only a point in time and hence cannot allow change to be observed over time. Although the sample size was relatively large, it may not represent the more significant pharmacist population. Moreover, the study’s focus on specific vaccines, such as tetanus and influenza, may not be generalizable to attitudes toward other vaccines. In addition, a lack of assessments of objective knowledge and in-depth qualitative insights limits the degree to which it is possible to understand the root causes of observed attitudes and knowledge gaps. Only 500 pharmacists submitted a survey out of the 6,100 who were sent the link, resulting in an 8.2% overall response rate. There is a high risk that the pharmacists who chose to participate (the “responders”) are inherently different from those who did not (the “non-responders”). Responders may have stronger opinions, greater knowledge, or greater interest in the topic of vaccination than the average pharmacist does, potentially leading to an over- or underestimation of key proportions. Although the internal consistency of the multi-item scales was acceptable (alpha = 0.727), the reliance solely on a self-developed, translated instrument, even after pilot testing, necessitates caution regarding the construct validity of the measured knowledge, attitudes, and barrier domains in the Turkish context.

## 6 Conclusion

The study identified critical knowledge gaps, particularly concerning tetanus vaccination and a complex, demographically influenced attitude landscape among community pharmacists in Turkey. Specifically, our data highlighted the influence of factors such as gender on vaccine safety perceptions and the inverse correlation between years of experience and perceived tetanus risk. We also confirmed significant systemic and logistical barriers, such as insufficient reimbursement and the widespread lack of mechanisms to identify unvaccinated patients. The primary goal of this research is to establish the necessary academic evidence base to inform effective policy and intervention strategies. The study unequivocally demonstrated that before fully advocating for the expansion of pharmacists’ scope of practice to include vaccine administration, substantial endeavors to improve their knowledge and attitudes and address current logistical hurdles must be prioritized. Ultimately, strengthening the internal capacity of the pharmacy workforce in tandem with advocating for legal and reimbursement reforms to grant pharmacists the authority to administer vaccines is essential for maximizing their contribution to public health outcomes in Türkiye. Future studies should explore the potential for growth in the role of pharmacists in immunization. By examining the impact of targeted educational enforcement and logistical support propositions in various neighborhoods, we can identify emerging barriers and facilitators. A longitudinal design would be particularly beneficial in this regard, offering a hopeful outlook on the evolution of pharmacists’ role in immunization.

## Data Availability

The original contributions presented in the study are included in the article/Supplementary material, further inquiries can be directed to the corresponding author/s.

## References

[1] Rodrigues CMC Plotkin SA. Impact of vaccines; health, economic and social perspectives. Front Microbiol. (2020) 11:1526. doi: 10.3389/fmicb.2020.01526, 32760367 PMC7371956

[2] Malande OO Munube D Afaayo RN Annet K Bodo B Bakainaga A . Barriers to effective uptake and provision of immunization in a rural district in Uganda. PLoS One. (2019) 14:e0212270. doi: 10.1371/journal.pone.0212270, 30763355 PMC6375600

[3] Paudyal V Fialová D Henman MC Hazen A Okuyan B Lutters M . Pharmacists’ involvement in COVID-19 vaccination across Europe: a situational analysis of current practice and policy. Int J Clin Pharm. (2021) 43:1139–48. doi: 10.1007/s11096-021-01301-7, 34218402 PMC8254632

[4] Arya S Norton N Kaushik P Brandtmüller A Tsoumani E. Recent changes to adult national immunization programs for pneumococcal vaccination in Europe and how they impact coverage: a systematic review of published and grey literature. Hum Vaccin Immunother. (2023) 19:2279394. doi: 10.1080/21645515.2023.2279394, 38014651 PMC10760380

[5] Cassimos D Effraimidou E Medić S Konstantinidis T Theodoridou M Maltezou HC. Vaccination programs for adults in Europe, 2019. Vaccine. (2020) 8:34. doi: 10.3390/vaccines8010034, 31968652 PMC7157239

[6] Haems M Lanzilotto M Mandelli A Mota-Filipe H Paulino E Plewka B . European community pharmacists practice in tackling influenza. Explor Res Clin Soc Pharm. (2024) 14:100447. doi: 10.1016/j.rcsop.2024.100447, 38707787 PMC11068921

[7] Poudel A Lau ETL DelDot M Campbell C Waite NM Nissen LM. Pharmacist role in vaccination: evidence and challenges. Vaccine. (2019) 37:5939–45. doi: 10.1016/j.vaccine.2019.08.060, 31474520

[8] Tadele S Demissie BN Tamiru MT Tadesse TA. Knowledge and attitudes of community pharmacists on vaccination, barriers and willingness to implement community pharmacy-based vaccination services in Ethiopia. Hum Vaccin Immunother. (2023) 19:2291243. doi: 10.1080/21645515.2023.2291243, 38111325 PMC10732657

[9] Balkhi B Aljadhey H Mahmoud MA Alrasheed M Pont LG Mekonnen AB . Readiness and willingness to provide immunization services: a survey of community pharmacists in Riyadh, Saudi Arabia. Saf Health. (2018) 4:2553. doi: 10.1186/s40886-018-0068-y

[10] Wakui N Kikuchi M Ebizuka R Yanagiya T Togawa C Matsuoka R . Survey of pharmacists' knowledge, attitudes, and practices (KAP) concerning COVID-19 infection control after being involved in vaccine preparation: a cross-sectional study. Int J Environ Res Public Health. (2022) 19:9035. doi: 10.3390/ijerph19159035, 35897405 PMC9331880

[11] Della Polla G Napolitano F Pelullo CP De Simone C Lambiase C Angelillo IF. Investigating knowledge, attitudes, and practices regarding vaccinations of community pharmacists in Italy. Hum Vaccin Immunother. (2020) 16:2422–8. doi: 10.1080/21645515.2020.1720441, 32048892 PMC7644221

[12] Ozdemir N Kara E Bayraktar-Ekincioglu A Buyukcam A Celiker A Demirkan K . Knowledge, attitudes, and practices regarding vaccination among community pharmacists. Prim Health Care Res Dev. (2022) 23:e38. doi: 10.1017/S1463423622000330, 35866296 PMC9309755

[13] Di Castri AM Halperin DM Ye L MacKinnon-Cameron D Kervin M Isenor JE . Healthcare provider awareness, attitudes, beliefs, and behaviors regarding the role of pharmacists as immunizers. Hum Vaccin Immunother. (2022) 18:2147356. doi: 10.1080/21645515.2022.2147356, 36472081 PMC9762776

[14] Quigley D Murphy KD. A qualitative evaluation of the attitudes, barriers, and facilitators relating to the provision of vaccine information by Irish community pharmacists. Int J Pharm Pract. (2022) 30:i17–7. doi: 10.1093/ijpp/riac021.023

[15] International Pharmaceutical Federation. An overview of current pharmacy impact on immunisation-a global report 2016. The Hague: International Pharmaceutical Federation (2016).

[16] Alkhudaidi SA Alqathami AM Alotaibi SR Alorabi MA Alosaimi NA. Pharmacy-based immunization services: expanding access and improving public health. Pharm Innov. (2024) 13:51–5. doi: 10.22271/tpi.2024.v13.i1a.25521

[17] Julia B Foerst C Akarkoub S Azzazene S Gremaud N Senegas RO . Expanding vaccination competencies to community pharmacists: modelling the organizational and economic impacts of new human papilloma virus (HPV) vaccine pathways in France. BMC Health Serv Res. (2024) 24:845. doi: 10.1186/s12913-024-11093-x, 39061059 PMC11282792

[18] World Health Organization. Process of translation and adaptation of instruments. Geneva: World Health Organization (2018).

[19] Pham T Peterson G Martin A Naunton M. Gender balance in Australian pharmacy organisations: are we there yet? Explor Res Clin Soc Pharm. (2024) 14:100442. doi: 10.1016/j.rcsop.2024.100442, 38707788 PMC11068630

[20] Hawthorne N Anderson C. The global pharmacy workforce: a systematic review of the literature. Hum Resour Health. (2009) 7:48. doi: 10.1186/1478-4491-7-48, 19545377 PMC2706790

[21] Chadi A Thirion DJG Waite NM David PM. Key stakeholder perspectives on delivery of vaccination services in Quebec community pharmacies. Can Pharm J (Ott). (2024) 157:304–14. doi: 10.1177/17151635241269988, 39539592 PMC11556574

[22] Dufour L Carrouel F Dussart C. Human papillomaviruses in adolescents: knowledge, attitudes, and practices of pharmacists regarding virus and vaccination in France. Viruses. (2023) 15:778. doi: 10.3390/v15030778, 36992485 PMC10058809

[23] Waszkiewicz M Wnuk K Świtalski J Augustynowicz A. Knowledge, attitudes, and beliefs of pharmacists regarding vaccinations against influenza and pneumococci – a systematic review. Hum Vaccin Immunother. (2025) 21:2489889. doi: 10.1080/21645515.2025.2489889, 40259436 PMC12013420

[24] Erku DA Belachew SA Abrha S Sinnollareddy M Thomas J Steadman KJ . When fear and misinformation go viral: pharmacists' role in deterring medication misinformation during the 'infodemic' surrounding COVID-19. Res Social Adm Pharm. (2021) 17:1954–63. doi: 10.1016/j.sapharm.2020.04.032, 32387230 PMC7252082

[25] Alyahya L Al-Ameri M Abu Farha R Mukattash T Noor DZA. Cross-sectional study of pharmacists’ knowledge and beliefs about human papillomavirus, its vaccines, and barriers related to vaccine administration. J Pharm Health Serv Res. (2024) 15:rmae016. doi: 10.1093/jphsr/rmae016

[26] Qamar M Koh CM Choi JH Mazlan NA. Community pharmacist’s knowledge towards the vaccination and their willingness to implement the community-based vaccination service in Malaysia. J Appl Pharm Sci. (2022) 1:128–39. doi: 10.7324/japs.2022.120612

[27] Ufuoma DA Adepeju OR Omuta MC. An appraisal of pharmacists’ involvement in immunization delivery in Lagos state, Nigeria. Res J Pharm Technol. (2023) 16:3201–6. doi: 10.52711/0974-360X.2023.00526

[28] Ariyo O Kinney O Brookhart A Nadpara P Goode JVKR. Medication therapy problems and vaccine needs identified during initial appointment-based medication synchronization visits. J Am Pharm Assoc. (2019) 59:S67–71. doi: 10.1016/j.japh.2019.04.019, 31153823

[29] Fava JP Colleran J Bignasci F Cha R Kilgore PE. Adolescent human papillomavirus vaccination in the United States: opportunities for integrating pharmacies into the immunization neighborhood. Hum Vaccin Immunother. (2017) 13:1844–55. doi: 10.1080/21645515.2017.1325980, 28605256 PMC5557233

[30] Pullagura GR Violette R Houle SKD Waite NM. Exploring influenza vaccine hesitancy in community pharmacies: knowledge, attitudes and practices of community pharmacists in Ontario, Canada. Can Pharm J. (2020) 153:361–70. doi: 10.1177/1715163520960744, 33282027 PMC7689625

[31] Pilbeam C Anthierens S Vanderslott S Tonkin-Crine S Wanat M. Methodological and ethical considerations when conducting qualitative interview research with healthcare professionals: reflections and recommendations as a result of a pandemic. Int J Qual Methods. (2022) 21:763. doi: 10.1177/16094069221077763

[32] Agbo BB Esienumoh E Inah SA Eko JE Nwachukwu EJ. Challenges in providing immunization services amongst community pharmacists in south-south, Nigeria: a cross-sectional study. J Adv Med Pharm. (2019) 21:1–8. doi: 10.9734/jamps/2019/v21i330133

[33] Hail A Abdulrouf P Stewart D Elkassem W Singh R Enany R . Behaviour and associated determinants of COVID-19 vaccine acceptance and advocacy: a nationwide survey of pharmacy professionals in Qatar. J Pharm Policy Pract. (2023) 16:160. doi: 10.1186/s40545-023-00668-4 38017533 PMC10683145

[34] Kumar R Alabdulla M Elhassan NM Reagu SM. Qatar healthcare workers' COVID-19 vaccine hesitancy and attitudes: a national cross-sectional survey. Front Public Health. (2021) 9:727748. doi: 10.3389/fpubh.2021.727748, 34513792 PMC8424093

[35] Kupferwasser D Flores E Merino P Tran D Bolaris M Gonzales M . Characterization of COVID-19 vaccine hesitancy among essential workforce members of a large safety net urban medical center. J Prim Care Community Health. (2023) 14:159814. doi: 10.1177/21501319231159814 PMC10028456 36941757

[36] Carvajal M Popovici I Hardigan PC. Gender differences in the measurement of pharmacists’ job satisfaction. Hum Resour Health. (2018) 16:33. doi: 10.1186/s12960-018-0297-5, 30064513 PMC6069841

[37] Radwan R Bentley J Patterson J Dixon D Salgado T. Predictors of job satisfaction among pharmacists: a regional workforce survey. Explor Res Clin Soc Pharm. (2022) 5:100124. doi: 10.1016/j.rcsop.2022.100124, 35478529 PMC9031680

[38] Ibrahim I Ibrahim M Majeed IA Alkhafaje Z. Assessment of job satisfaction among community pharmacists in Baghdad, Iraq: a cross-sectional study. Pharm Pract. (2021) 19:2190. doi: 10.18549/pharmpract.2021.1.2190, 33777262 PMC7979314

[39] Taylor S Mylrea M Eastaughffe J Dixon R Kent I Kappel C . Rural pharmacist and consumer perspectives of expanded pharmacy services to address inequity in accessing health services. Int J Pharm Pract. (2025) 33:73–80. doi: 10.1093/ijpp/riae061, 39527573

[40] Chu J Maharajan M Rajiah K. Perspectives of community pharmacists on extended pharmacy services and value-added services in Malaysia: a cross-sectional survey. Int J Pharm Pract. (2024) 32:146–55. doi: 10.1093/ijpp/riad087, 38071745

[41] Jarab A Al-Qerem W Alzoubi K Almomani N Heshmeh S Mukattash T . Exploring pharmacists’ attitude, willingness and barriers to provide extended community pharmacy services: implications for improved pharmacy services. PLoS One. (2024) 19:e0310141. doi: 10.1371/journal.pone.0310141 39250510 PMC11383215

[42] Sabra R Safwan J Dabbous M Rida A Malaeb D Akel M . Assessment of knowledge, attitude and practice of Lebanese pharmacists in providing patient counseling on urinary tract infection and its treatment. Pharm Pract. (2022) 20:01–10. doi: 10.18549/pharmpract.2022.2.2653, 35919798 PMC9296090

[43] Vigneshwaran E Shahrani S Alanzi A Mohammad A Sadiq M Khan N . Comparing knowledge, attitudes, and practices in cardiovascular disease prevention and health promotion between community and hospital pharmacists in Saudi Arabia: a cross-sectional study. Saudi Pharm J. (2024) 32:101890. doi: 10.1016/j.jsps.2023.101890 38192383 PMC10772382

[44] Avramidis I Pagkozidis I Domeyer P Papazisis G Tirodimos I Dardavesis T . Exploring perceptions and practices regarding adult vaccination against seasonal influenza, tetanus, pneumococcal disease, herpes zoster and COVID-19: a mixed-methods study in Greece. Vaccine. (2024) 12:80. doi: 10.3390/vaccines12010080, 38250893 PMC10818817

[45] Byrne H Hew H Mullally T O’Malley F Orimolusi T Tiarnaigh KÁ . Vaccination services in community pharmacy practice: a scoping review. Int J Pharm Pract. (2024) 32:i35–6. doi: 10.1093/ijpp/riae013.044

[46] Jarab AS Al-Qerem W Mukattash TL. Community pharmacists' willingness and barriers to provide vaccination during COVID-19 pandemic in Jordan. Hum Vaccin Immunother. (2022) 18:2016009. doi: 10.1080/21645515.2021.2016009, 35050841 PMC8986174

[47] Kulczycki A Hogue M Rothholz M Grubbs J Shewchuk R. 2022 national survey of immunizing community chain pharmacists: the case for codifying the PREP act emergency authorities into practice. J Am Pharm Assoc. (2024) 64:79–87. doi: 10.1016/j.japh.2023.10.016, 37863397

[48] Thidrickson D Goodyer L. Pharmacy travel health services in Canada: experience of early adopters. Pharmacy. (2019) 7:42. doi: 10.3390/pharmacy7020042, 31035567 PMC6631395

[49] Merks P Religioni U Bilmin K Lewicki J Jakubowska M Waksmundzka-Walczuk A . Readiness and willingness to provide immunization services after pilot vaccination training: a survey among community pharmacists trained and not trained in immunization during the COVID-19 pandemic in Poland. Int J Environ Res Public Health. (2021) 18:599. doi: 10.3390/ijerph18020599, 33445750 PMC7828205

[50] Kummer GL Foushee LL. Description of the characteristics of pharmacist-based immunization services in North Carolina: results of a pharmacist survey. J Am Pharm Assoc. (2003) 2008:744–51. doi: 10.1331/JAPhA.2008.07080, 19019803

[51] Edwards N Corsten EG Kiberd M Bowles S Isenor J Slayter K . Pharmacists as immunizers: a survey of community pharmacists' willingness to administer adult immunizations. Int J Clin Pharm. (2015) 37:292–5. doi: 10.1007/s11096-015-0073-8, 25687902

[52] Van Amburgh JA Waite NM Hobson EH Migden H. Improved influenza vaccination rates in a rural population as a result of a pharmacist-managed immunization campaign. Pharmacotherapy. (2001) 21:1115–22. doi: 10.1592/phco.21.13.1115.34624, 11560201

[53] Bayraktar-Ekincioglu A Kara E Bahap M Cankurtaran M Demirkan K Unal S. Does information by pharmacists convince the public to get vaccinated for pneumococcal disease and herpes zoster? Ir J Med Sci. (2022) 191:2193–200. doi: 10.1007/s11845-021-02778-x, 34708289 PMC8550811

